# Performance Comparison of Argos and Iridium Tracking Technologies for Sea Turtle Movement Ecology Studies

**DOI:** 10.3390/ani15243605

**Published:** 2025-12-15

**Authors:** Paolo Casale, Christine Figgener, Michael Arendt, Annette C. Broderick, Simona A. Ceriani, Yakup Kaska, Pamela Plotkin, Cheryl L. Sanchez, Jeffrey Schwenter, Robin Snape, Doğan Sözbilen, Natalie E. Wildermann, Paolo Luschi

**Affiliations:** 1 Department of Biology, University of Pisa, Via A. Volta 6, 56126 Pisa, Italy; 2 Namaka Conservation Science, 70402 Gandoca, Talamanca, Sixaola, Limón, Costa Rica; 3 Department of Natural Resources, Marine Resources Division, Charleston, SC 29412, USA; 4 Centre for Ecology and Conservation, University of Exeter, Penryn Campus, Truro TR10 9FE, UK; 5 Fish and Wildlife Research Institute, Florida Fish and Wildlife Conservation Commission, St. Petersburg, FL 33701, USA; 6 Department of Biology, Faculty of Science, University of Pamukkale, Pamukkale, Denizli 20070, Türkiye; 7 Department of Oceanography, Texas A&M University, College Station, TX 77843, USA; 8 Society for the Protection of Turtles (SPOT), Barbaros sok (23/2), Gönyeli, 99150 Nicosia, North Cyprus; 9 Department of Veterinary, Acıpayam Vocational School, Pamukkale University, Denizli 20070, Türkiye; 10 Marine Science Program, Division of Biological and Environmental Science and Engineering, King Abdullah University of Science and Technology, Thuwal 23955, Saudi Arabia

**Keywords:** marine megafauna, GPS, satellite telemetry, animal-borne sensors, Bayesian modelling, spatiotemporal resolution

## Abstract

Satellite tracking has dramatically improved research on wide-ranging large marine vertebrates such as sea turtles. Traditionally, sea turtle tracking has relied on Argos-based satellite telemetry tags, which estimate location via Doppler shift and can also transmit sensor data. GPS-equipped Argos satellite tags represented a significant evolution, offering higher location accuracy. More recently, GPS-equipped satellite tags transmitting via the Iridium satellite network have become available for sea turtle tracking, and this study aims to assess whether they offer additional advantages. The performance of three satellite tag types—Argos-only, Argos-GPS, and Iridium-GPS (Iridium)—was assessed using data on 116,074 positions from 48 sea turtles representing five species and multiple ocean basins. Performance was evaluated using three indicators: the proportion of days with location data, the duration of gaps between locations, and the number of positions per day. Bayesian generalized linear mixed models assessed the effect of satellite tag type, technical settings, species, and activity (migration, foraging, internesting). Results indicate that Iridium satellite tags generally perform similarly to both Argos-based satellite tags, but performance improves significantly when programmed with high-frequency GPS acquisition (>24 positions/day), a result made possible by their tenfold higher transmission capacity compared to Argos. This capacity also enables transmission of more sensor data. Performance, however, varied by species and activity. These findings highlight the potential of Iridium tags to enhance fine-scale movement studies by improving the spatial and temporal resolution of sea turtle tracking, with important implications for ecological research and conservation planning.

## Simple Summary

Understanding where sea turtles go and how they use the ocean is essential for protecting them, but following their movements is challenging because they travel long distances and spend most of their time underwater. Scientists use satellite tags attached to turtles’ shells to collect location data when they surface. For many years, these tags have transmitted data through the Argos satellite system, which provides useful but sometimes limited information. A newer satellite network, called Iridium, has different and possibly useful features. In this study, we compared three types of tracking tags—two using the Argos system and one using Iridium—by analyzing over 100,000 locations from 48 turtles of five species in different oceans. We found that Iridium tags performed as well as the Argos ones overall, and even better when programmed to record positions more frequently. Because Iridium can send much more data, it allows researchers to study turtle movements in finer detail. This improvement will help scientists better understand turtle behavior, migration, and the threats they face, ultimately supporting more effective conservation efforts.

## 1. Introduction

Animal-borne devices linked to satellite systems have dramatically improved the research on large marine vertebrates, which typically move across vast areas [1]. For sea turtles, satellite tracking began in the 1980s [2] and has since enabled investigation of fundamental aspects of their biology, in particular their distribution (e.g., nesting and foraging areas, migratory corridors) [3,4,5,6,7,8] which can also identify hot-spot areas where conservation measures should be most effective [9,10,11,12,13,14,15,16]. Satellite tracking also contributed to other fundamental aspects such as breeding frequency [5,6,7,17,18,19,20], movement patterns [21,22], migratory patterns and connectivity [9,23,24,25].

Traditionally, satellite tracking has been conducted through enhanced radio-transmitters, hereafter referred to as satellite telemetry tags linked to the Argos satellite constellation orbiting ~850 km above Earth’s surface. Since the inception of the Argos system in 1978, the number of operational satellites has fluctuated, stabilizing at approximately six to seven in the past decade (https://cnes.fr/en/projects/argos; accessed on 28 September 2025). These receivers process radio waves emitted from satellite tags and in turn estimate satellite tag location anywhere on Earth. Position reckoning is complex and based on measurements of the Doppler shift between signals received for at least two successive transmissions of the satellite tag during a single overpass of the same satellite (https://www.argos-system.org/using-argos/how-argos-works/; accessed on 28 September 2025). As for other methods employing radio signals, the process works only with aerial transmissions, and in sea turtles is limited to the unevenly spaced and somewhat infrequent surfacing events, when the turtle surfaces for a short time (usually seconds) to breathe [26]. Location accuracy reflects the number of satellite tag signals received within a single satellite overpass, and is characterized using six location classes (LCs: 0, 1, 2, 3, A, and B). Error radii for LCs 0, 1, 2, and 3 range from <150 to >1000 m [www.argos-system.org]; [27,28,29].

However, typical satellite tag locations encountered in sea turtle studies are often LCs A and B, with no precise error estimate, but which have been found to be accurate to 3.5 ± 9.2 km and 14.3 ± 135.6 km of the true position, respectively [27]. Thus, LCs A and B are not considered suitable for informing fine-scale marine spatial planning [30]. Satellite tag transmissions may also contain data recorded by sensors that measure environmental (e.g., water temperature) or behavioral (e.g., dive durations or frequency, or even dive depth profiles) metrics. A significant limitation of Argos satellite tags is that many sea turtle populations inhabit tropical waters close to the equator, where satellite overflights are limited to a few per day due to the polar-orbiting nature of the constellation, resulting in several hours per day without a single satellite in view.

The low location accuracy of traditional Argos locations and the limited satellite tag capacity to transmit data led to the development of a new satellite tag that integrated a Global Positioning System (GPS) receiver in an Argos transmitter. While conventional GPS in terrestrial applications may provide accurate data at low cost [30], for marine animals that surface only briefly, special technologies are needed to rapidly acquire GPS data and allow accurate positions and different manufacturers use different techniques (e.g., Fastloc^®^, FastGPS or Quickfix).

GPS-Fastloc^®^ technology is particularly useful, as it can rapidly (<100 milliseconds) acquire accurate positions. For instance, in sea turtles, 50% of positions have been reported to have an error within 36 m or 18 m, depending on the number of GPS satellites received [31]. The GPS data are then transmitted through the Argos connection, along with other data from sensors. The narrow bandwidth of Argos transmissions (maximum message length 256 bits) limits the data transmission capacity of such Argos-GPS satellite tags—including any sensor data—but obtaining high-quality GPS locations represented a great improvement for sea turtle studies focusing on fine-scale movements, where high spatial accuracy and temporal frequency are needed [31,32,33,34].

Recently, GPS satellite tags linked to the Iridium satellite constellation (Iridium satellite tag) have become commercially available and are being used in sea turtle studies [16,24,35,36]. These tags are comparable in size to the Argos-linked models, but they lack an external antenna. The Iridium system was established in 1998 for satellite phone communication and consists of 66 low polar-orbiting satellites that provide continuous global coverage. Satellite tags linked to the Iridium constellation and deployed on sea turtles are manufactured by Telonics Inc., which currently is the only company producing Iridium tags suitable for marine animals. They obtain GPS positions through Quick Fix Pseudoranging (QFP) technology (calculating a position in 2–3 s; https://www.telonics.com/products/gps4Marine/seatrkr.php; accessed on 28 September 2025), with accuracy < 11 m [16]. They then relay these locations to the Iridium satellites along with other data acquired by sensors onboard. Two notable aspects of the Iridium network provide, in theory, important advantages for wildlife tracking compared to Argos: (a) each signal can encode more data than Argos (340 vs. 32 bytes) and (b) Iridium satellites provide a continuous worldwide coverage while Argos satellites currently do not. These features have the potential to significantly enhance the quality of data collected for air-breathing, marine species such as sea turtles. While the performance of Argos and Argos-GPS satellite tags has been previously compared [31], here we investigate whether Iridium satellite tags can offer better performance than Argos-only and Argos-GPS satellite tags, using a large dataset assembled across sea turtle species and global study sites.

## 2. Materials and Methods

### 2.1. Data Collection

We analyzed data originating from 48 satellite tags attached to the carapace of individual sea turtles (as part of separate local projects, each authorized by the relevant national authority for turtle tagging procedures) and linked to either the Argos (*n* = 22) or Iridium (*n* = 26) satellite constellations (Supplementary Table S1). All Iridium satellite tags were model SeaTrkr-4370-4 by Telonics Inc. (Mesa, AZ, USA). Argos satellite tags were model TAM-4310-3 (Telonics; *n* = 7), SPLASH (Wildlife Computers, Redmond, WA, USA; *n* = 6), MK10 (Wildlife Computers; *n* = 4) and F6G276F (Lotek, Newmarket, ON, Canada; *n* = 5) (Supplementary Table S1). The latter 15 Argos satellite tags (all except the seven Telonics devices) also had a fast-acquisition GPS receiver (Fastloc^®^ GPS for Wildlife Computers devices and FastGPS for Lotek devices) and therefore provided both Argos and GPS-derived positions, which we analyzed separately. For Argos, all LCs were used; however, we excluded the 1-message B positions that were estimated by Argos through interpolation of only one message with previous data. For Iridium satellite tags we considered only GPS positions, even though the satellite tags can also provide Doppler-based locations similar to those of Argos. This choice reflects the fact that transmissions to the Iridium satellites are made (and so Doppler-based locations can be obtained) only when the unit is ready to send a complete data package stored in its memory, and so much less frequently than in Argos satellite tags. Therefore, we considered three types of position data for comparison: Iridium, ARGOS-GPS and Argos-derived positions (ARGOS). Some satellite tags provided both ARGOS-GPS and ARGOS positions and we analyzed these positions separately, according to their type. Since both Argos and Iridium satellites follow polar orbits, communication with ground-based transmitters is suspected to occur less frequently at lower latitudes, due to fewer satellite overpasses per day, in particular for the Argos constellation, which consists of a relatively low number of satellites and a low number of orbital planes. Therefore, latitude might affect both ‘constellations’ performance and, for this reason, we included latitude in the analyses (see below).

Satellite tag programming encompasses a range of settings, three of which we considered particularly important when evaluating performance. First, most satellite tags were programmed to transmit when at the sea surface (i.e., no duty cycle); however, nine Argos satellite tags out of 48 satellite tags had a duty cycle (less than 24 h on), which saves battery life and can be employed when satellite overflights over certain regions are not to be expected at a specific time of day. However, duty cycles can affect the number of Argos positions obtained, given that Argos position quality is contingent on the number of transmissions received; therefore, duty cycle was considered in the analyses (see below). Second, different GPS satellite tags (types ARGOS-GPS and Iridium) were programmed to determine a GPS position at a different rate (e.g., every three or six hours). From this rate, we calculated the theoretical maximum number of positions per day (MPD) and we considered it as a potential scenario affecting the number of Iridium and ARGOS-GPS positions. Third, Iridium satellite tags transmit a data package only when their 340-byte package is full or after a specific time (e.g., 24 or 48 h) since the last transmission even if the data package is not full (Iridium maximum transmission interval, IMTI). However, since transmissions may not be successful and Iridium satellite tags give priority to the most recent data, some data packages may be lost if transmissions fail for an extended period and too many packages accumulate. Therefore, we considered IMTI as another potential parameter affecting the number of Iridium positions. Some satellite tags were programmed to change MPD settings after a specific date; thus, we considered periods with different settings of the same turtle/satellite tag separately.

All data originated from the Northern Hemisphere (latitudes 9° N through 37° N), except for three tracks from the Indian Ocean (−9° S, Supplementary Table S1), and spanned five bodies of water (ordered north to south): Mediterranean Sea (*n* = 11), Northwest Atlantic Ocean (*n* = 13), Gulf of Mexico (*n* = 12), Northeast Pacific Ocean (*n* = 6), and Caribbean Sea (*n* = 3). Irrespective of geography, our data included tracks from five sea turtle species: *Caretta caretta* (*n* = 23), *Chelonia mydas* (*n* = 12), *Lepidochelys olivacea* (*n* = 6), *Lepidochelys kempii* (*n* = 4), and *Eretmochelys imbricata* (*n* = 3) (Supplementary Table S1). Turtles were captured on nesting beaches, captured in-water during research surveys, incidentally captured by fishing gear or were rehabilitated (Supplementary Table S1). Authors classified their respective data according to one of three activity types (inter-nesting, i.e., during the nesting period, migration, or foraging; Supplementary Table S1), which were analyzed separately.

### 2.2. Data Analysis

We aggregated data by date (day) from the deployment to the end of tracking, including days with no transmissions or positions. For each day, we calculated three response variables and six predictor variables as follows.

We investigated the performance of the different satellite tags through three performance indicators (response variables): the proportion of days with at least one position (days with position, DP), the interval (in days) with no positions between two consecutive days with at least one position (zero positions interval, ZPI), and the number of positions within the same day (number of positions per day, NPD). The first two indicators (DP and ZPI) helped us investigate the suitability of satellite tags for studies on coarse-scale movements, where periods without positions represent a vital information gap within a turtle track. The third indicator (NPD) allowed us to investigate the suitability of satellite tags for studies on fine-scale movements, where the critical difference is represented by the frequency of positions at short time intervals. For each day, we calculated DP and NPD values. Then, we assigned a ZPI value to the last day of each series of days with no positions.

We considered as biological and technical covariates a total of six predictor variables (three categorical and three numerical): the satellite tag type (categorical; 3 levels: ARGOS, ARGOS-GPS and Iridium, see above), turtle species (categorical; five levels, see above), turtle Activity (categorical; three levels, see above), maximum number of GPS positions per day (numerical; MPD, see above; for ARGOS-GPS and Iridium satellite tags only), Iridium maximum transmission interval (numerical; IMTI, see above; for Iridium satellite tags only), and latitude (numerical). For each day, we assigned to that day the latitude of the first position; if no positions were available, we used the most recent previously known latitude instead. We used absolute latitude values (i.e., southern latitudes were converted to positive values).

We investigated the effects of predictors on the response variables through Bayesian generalized linear mixed models (BGLMM) with the individual turtle as a random factor. We ran BGLMMs through the package brms [37] in R [38]. Priors for regression coefficients were left at the default settings used in brms, which are weakly informative to regularize estimates and prevent overfitting. For the predictor satellite tag type, we set Iridium as the reference level because it was the focus of the study. For the other two categorical predictors (species and activity), we set the most abundant level as the reference level. We assessed model convergence using the Gelman-Rubin diagnostic (R-hat < 1.01) and effective sample sizes (i.e., Bulk Effective Sample Size (Bulk_ESS) and Tail Effective Sample Size (Tail_ESS) > 400), indicating adequate mixing of the chains. We assessed model fit via the Bayesian *p*-value (the proportion of posterior predictive means that are greater than or equal to the observed mean), with values close to 0.5 indicating good fit while values near 0 or 1 suggest lack of fit [39]. We considered a predictor meaningful if the 95% Credible Intervals (rounded to two decimal places) did not include zero. For each model, we evaluated collinearity among predictors using Variance Inflation Factors (VIFs), calculated with the vif() function from the car package, applied to a linear model fitted using the lm() function from the stats package with a constant response. This approach allowed assessment of collinearity in the fixed-effects design matrix independently of the model family or random effects. A VIF value below five was considered indicative of a lack of problematic collinearity.

To examine the effect of different main predictors on the three response variables, we used two datasets, resulting in a total of six models (Table 1). First, we used a dataset including only Iridium data to investigate the effect of MPD and IMTI on this type of satellite tag. Then, we used a second dataset including all three satellite tag types (ARGOS, ARGOS-GPS and Iridium) to investigate the effect of satellite tag type. With this dataset, to account for the effect of duty cycle (DC), we used only ARGOS data with no duty cycle in models with DP or ZPI as response variables, while in the model with NPD as response variable, we calculated a corrected NPD value for ARGOS data with DC < 24: NPD_corr_ = NPD/DC*24. In addition to comparing ARGOS and ARGOS-GPS with Iridium (reference level) through the standard BGLMM results, we also compared ARGOS and ARGOS-GPS through the hypothesis() function of the brms package. We included only Iridium records with IMTI = 24 because of the suspected (and also observed, see results) negative effect of a higher IMTI value on the performance. In all models with NPD as the response variable, we used only records with NPD > 0 because the number of positions in a day with positions was of interest here while the number of days with or without positions was investigated through the other two response variables (DP and ZPI).

## 3. Results

**Figure 1:**
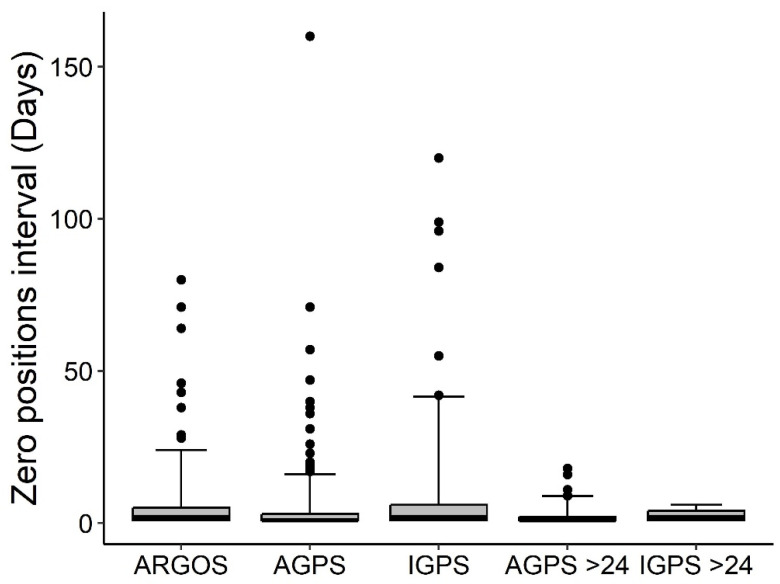
Duration (days) of intervals without any positions (zero positions interval, ZPI) by satellite tag type: ARGOS (*n* = 380), ARGOS-GPS (*n* = 599), Iridium (*n* = 206). Only ARGOS data with no duty-cycle (24/24 h active) were included. Subsets with the theoretical (from satellite tag settings) maximum GPS positions per day (MDP) > 24 are also shown for comparison (ARGOS-GPS > 24, *n* = 123; Iridium > 24, *n* = 17). Boxplots: median, 50% percentile (line), 25–75% (box) and 95% percentile range (whiskers), outliers (dots).

**Figure 2:**
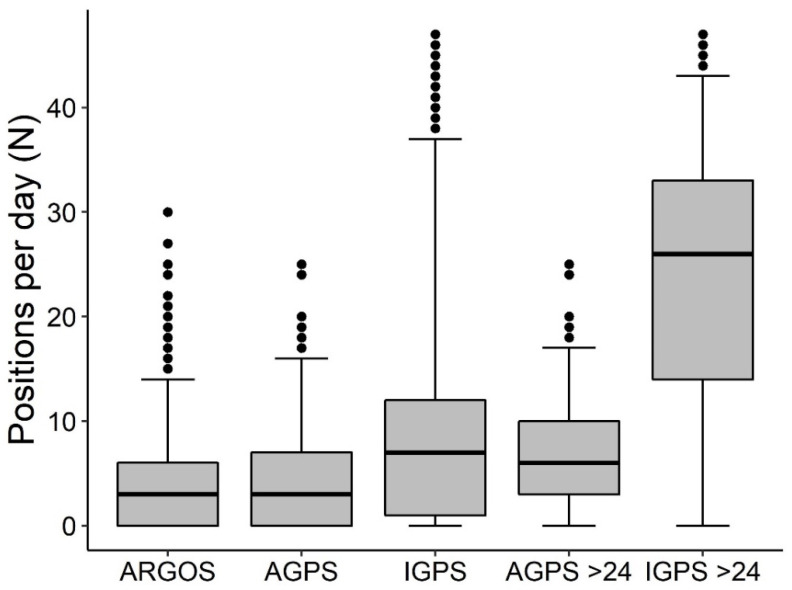
Number of positions per day (NPD) by satellite tag type: ARGOS (*n* = 5909), ARGOS-GPS (*n* = 5984), Iridium (*n* = 6327). Only ARGOS data with no duty-cycle (24/24 h active) were included. Subsets with the theoretical (from satellite tag settings) maximum GPS positions per day (MDP) > 24 are also shown for comparison (ARGOS-GPS > 24, *n* = 2956; Iridium > 24, *n* = 1029). Boxplots: median, 50% percentile (line), 25–75% (box) and 95% percentile range (whiskers), outliers (dots).

**Figure 3:**
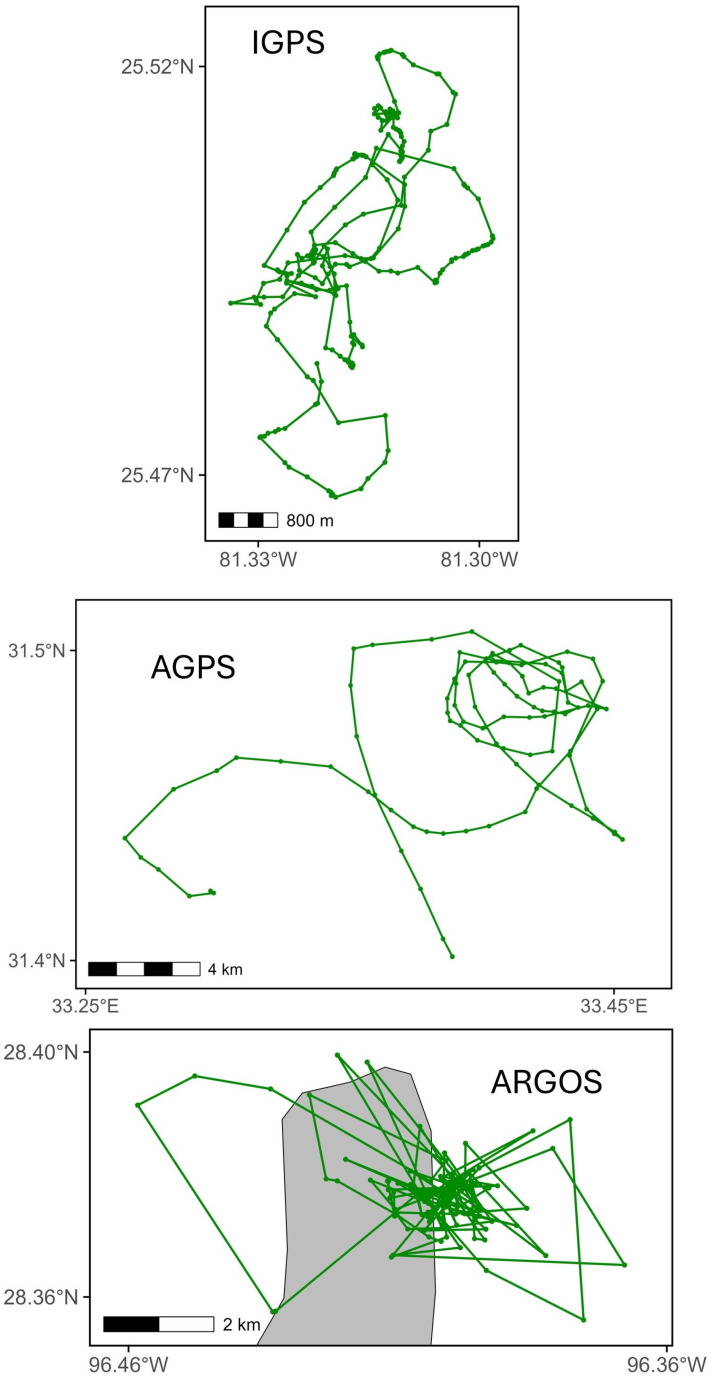
Examples of tracks with the highest number of positions per day for each type of satellite tag, during six consecutive days. Iridium GPS (Iridium): turtle #16 (*Caretta caretta*, Florida, USA, Gulf of Mexico; 12–17 August 2020; *n* = 254); Argos GPS (ARGOS-GPS): turtle #11 (*Caretta caretta*, Cyprus, Mediterranean; 5–10 July 2019; *n* = 98); ARGOS: turtle #26 (*Chelonia mydas*, Texas, USA, Gulf of Mexico; 2–7 July 2021; *n* = 121). Gray areas represent land.

A total of 116,074 positions were recorded over 19,035 tracking days across six marine areas and five sea turtle species (Table 2). Table 3 summarizes results aggregated by activity and satellite tag type (Supplementary Table S1 provides detailed results by turtle, satellite tag type, activity, and settings). Among the three performance indicators, days with at least one position (DP) predominated, while days with no positions accounted for 21.0% in Iridium, 30.2% in ARGOS-GPS, and 30.5% in ARGOS satellite tags (considering only ARGOS units without a duty cycle). The median zero-position interval (ZPI) across days with no positions was 2 days (IQR = 5; *n* = 206) for Iridium, 1 day (IQR = 2; *n* = 599) for ARGOS-GPS, and 2 days (IQR = 4; *n* = 380) for ARGOS (Figure 1). In the subset with MDP set at >24, ZPI values were 2 days (IQR = 3; *n* = 17) for Iridium and 1 day (IQR = 1; *n* = 123) for ARGOS-GPS (Figure 1). The median number of positions per day (NPD) was 7 (IQR = 11; *n* = 6327) for Iridium, 3 (IQR = 7; *n* = 5984) for ARGOS-GPS, and 3 (IQR = 6; *n* = 5909) for ARGOS (Figure 2). In the subset with MDP >24, median NPD increased to 26 (IQR = 19; *n* = 1029) for Iridium and 6 (IQR = 7; *n* = 2956) for ARGOS-GPS (Figure 2). Examples of tracks reconstructed from the three types of satellite tags illustrate the potentially better performance of Iridium tags with MDP > 24 compared to ARGOS-GPS and ARGOS tags (Figure 3). The median interval between two consecutive transmissions of Iridium tags was 7.5 h (95% quantiles: 0.0–72.5 h).

**Figure 4:**
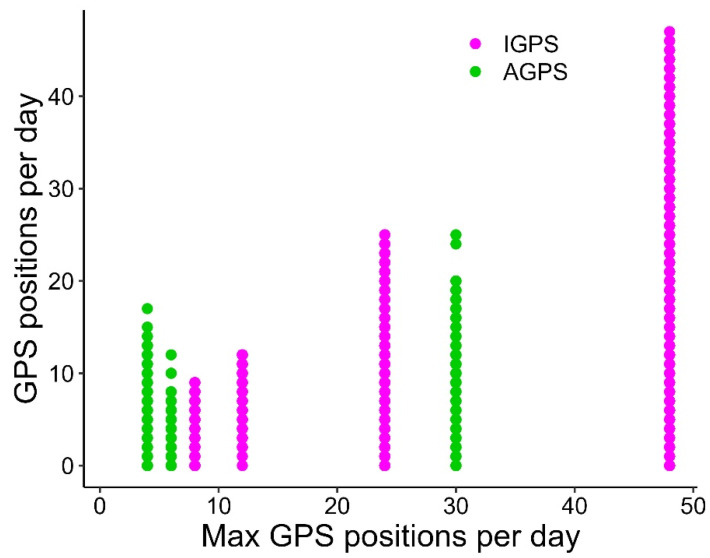
Positive relationship (see text) between the observed number of positions per day (NPD) and the theoretical (from satellite tag settings) maximum number of GPS positions per day (MPD; 4, 6, 8, 12, 24, 30, 48; see Supplemental Table S1) from 26 turtles equipped with Iridium-GPS satellite tags (Iridium; *n* = 6327). Data from 15 turtles equipped with Argos-GPS satellite tags (ARGOS-GPS; *n* = 5984) are also shown for comparison.

**Figure 5:**
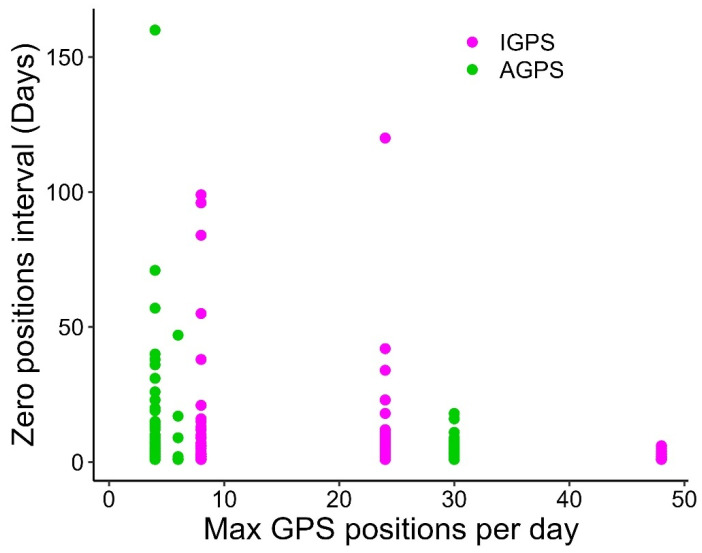
Negative relationship (see text) between the observed duration (days) of intervals (*n* = 805) without any position (ZPI) and the theoretical (from satellite tag settings) maximum number of GPS positions per day (MPD; 4, 6, 8, 24, 30, 48; see Supplemental Table S1) from 27 turtles equipped with Iridium-GPS satellite tags (Iridium; *n* = 206) (other 9 turtles with GPS satellite tags had no ZPI, i.e., they provided at least 1 position every day). Data from 15 turtles equipped with Argos-GPS satellite tags (ARGOS-GPS; *n* = 599) are also shown for comparison.

Models based on Iridium data (Table 1) indicated that the maximum transmission interval (IMTI) had a credible positive effect on the duration of periods with no positions (ZPI), and a suspected effect (i.e., the credible interval did not entirely exclude zero) on the number of days with at least one position (DP). In contrast, the maximum transmission interval (IMTI) had no credible effect on the number of positions per day (NPD). The theoretical maximum number of positions per day (MPD) showed a credible positive effect on both the proportion of days with at least one position (DP) and the number of positions per day (NPD) (Figure 4), and produced a credible negative effect on zero position interval (ZPI), i.e., shorter ZPIs (Figure 5). Accordingly, MPD greatly enhances the performance of Iridium tags in comparison to the other types (Figure 1 and Figure 2). However, according to the model results, Iridium tags did not exhibit an overall performance different from the other two satellite tag types, while ARGOS showed longer zero position intervals (ZPI) than ARGOS-GPS tags (Table 1).

The performance of satellite tags was affected by both species and activity (Table 1). With Iridium satellite tags, *Lepidochelys olivacea* and *Eretmochelys imbricata* showed a higher number of days with at least one position (DP) than *Caretta caretta* (the reference species). *Chelonia mydas* showed lower performance in terms of both the number of days with at least one position (DP) and the number of positions per day (NPD) compared to *C. caretta*, whereas the opposite pattern was observed for the number of days with at least one position (DP) when all satellite tag types were considered. *Lepidochelys kempii* showed higher performance in terms of ZPI when all satellite tag types were considered. With Iridium satellite tags, performance decreased during inter-nesting (DP, ZPI, and NPD) and migration (DP and NPD) relative to foraging (the reference activity), while the opposite trend was observed when all satellite tag types were considered. The hypothesized positive effect of latitude on performance was observed only for the number of days with at least one position (DP) and ZPI, while a negative effect on performance was detected for the number of positions per day (NPD) (all satellite tags). In Iridium satellite tags only, latitude had a negative effect on performance for both DP and NPD (Table 1).

## 4. Discussion

Our results indicate that Iridium satellite tags generally do not perform differently from the other two Argos-based satellite tag types when comparing solely the three performance indicators we chose for our analysis. Although Iridium data in our dataset start in 2017 and some Argos data precede that year, it is unlikely that any temporal effect would mask a real difference between tag types. However, the performance of Iridium tags can significantly improve—and greatly surpass that of the Argos-based satellite tags—when they are programmed to collect a high number of GPS positions per day (MPD), notably more than 24. Under these conditions, not only would the number of positions per day (NPD) increase, but also the number of days with at least one position (DP), while the number of consecutive days with no positions (ZPI) would decrease. The tenfold higher transmission capacity of Iridium satellite tags compared to Argos satellite tags is the key factor enabling the transmission of a larger number of positions and overall better performance. Although a high MPD also improves the performance of ARGOS-GPS, the lower transmission capacity of the Argos system limits this effect. Results also show that setting the minimum transmission interval (IMTI) of Iridium satellite tags to > 24 h may be detrimental, likely because some data packages never get transmitted. Future improvements of the Argos network—in terms of the number of satellites (https://kineis.com/en/high-five-complete-constellation-mission-accomplished; https://wildlifecomputers.com/blog/important-update-satellite-service-changes-and-the-future-of-your-data; both accessed on 28 September 2025)—will likely reduce periods without satellites able to receive signals from Argos satellite tags. The effect of latitude on satellite tag performance is not clear, with contrasting effects depending on the indicator considered, and latitude showed a negative effect on the performance of Iridium tags, contrary to expectations.

While Iridium tags may offer no advantage for studies requiring only coarse-scale movement data, their potential performance can enable the detection of fine spatiotemporal-scale movement data that would otherwise go unnoticed (Figure 3). For example, Iridium tags can facilitate investigations of research topics that have so far been difficult to address, such as high-resolution identification of internesting or foraging areas (e.g., [24]) and fine-scale movements during migration in relation to ocean currents and orientation mechanisms (e.g., [34,35,40]).

The higher transmission capacity of Iridium tags also allows researchers to obtain more detailed sensor data (e.g., dive profiles) than is possible through the Argos network. On the other hand, high MPD values will increase the data volume transmitted by Iridium tags, leading to a faster battery drain and shorter satellite tag longevity (as well as to an increase in tracking costs; see below). During device setup, a trade-off could then arise between data volume needs and the long duration of tracking, especially in the case of smaller satellite tags that have lower battery capacity. However, current Iridium tags include two-way communication, which allows remote modification of the unit’s programming after deployment so transmission frequency may be reduced to prolong tracking. Another positive feature is the absence of an external antenna, which eliminates the risk of antenna breakage—a common cause of tag failure [41]—and may also reduce the risk of entanglement in high-risk areas. That said, the internal antenna may result in slightly longer transmission times between satellite tag and the satellite, and may cause issues where animals do not surface as often or for prolonged periods. In contrast, ARGOS-GPS satellite tags benefit from a considerably faster connection to the satellites, often occurring within milliseconds, making them better suited in places where surfacing periods are expected to be very short.

Further, our data strongly suggest that turtle species and activity may affect all types of satellite tags, but in different ways. This is an aspect to consider when planning a study, as the advantage of using a specific satellite tag type may be offset by disadvantages related to the target species and activity. For instance, results indicate that Iridium satellite tags perform better for *Caretta caretta* than *Chelonia mydas,* and better during foraging than during inter-nesting or migration. The opposite trends are suggested for Argos-based satellite tags. Given the longer time required by Iridium tags than ARGOS-GPS tags to relay a GPS position to satellites, we hypothesize that these differences may reflect different breathing patterns between species and activity types. Results for the other species are less conclusive, likely due to their underrepresentation in the dataset. Although the two fast GPS acquisition systems used in our satellite tags (Fastloc^®^ and FastGPS) may have slightly different acquisition times, this aspect does not affect our conclusions, because no difference was found between ARGOS-GPS and Iridium tags, despite the different acquisition times of Iridium tags.

An issue that is often pivotal in determining choices of satellite tags is the cost of units and data transmission. The cost of tags is highly variable, depending on the type of tags and on their performances (e.g., battery duration, presence of depth sensors and so on). Argos-only units are typically the cheapest, with some simple models costing around 1000 EUR, while GPS satellite tags (both Argos and Iridium) are 2–3 times the price. The cost of satellite services, too, is quite variable, depending on tag settings and performances (e.g., number of contacts with satellites, amount of data relayed and so on). Wildlife tracking through Argos benefits from special fees that lead to a maximum monthly cost of around 65 EUR/tag, while Iridium fees are somewhat lower (around 25–35 EUR/tag for the two plans most commonly used for turtle tracking, according to metOcean price list). However, the actual costs may vary substantially depending on each tag’s performance during the period of tracking.

## 5. Conclusions

Iridium GPS satellite tags can provide similar performance to Argos GPS satellite tags and potentially better performance if programmed with a high frequency of GPS acquisition (>1 h^−1^) and transmission frequency ≥ 1 day^−1^. However, this may not apply to all species or activity types. Iridium GPS satellite tags could enable studies at fine spatiotemporal scales that have rarely been undertaken so far, with significant implications for ecological research and conservation planning.

## Figures and Tables

**Table 1:** Settings and results of six Bayesian GLMMs. Symbol + (−) in numerical variables indicates a credible positive (negative) relationship between the predictor and the response variable; in categorical variables it indicates that the shown level has a credible increase (decrease) in the response variable relative to the reference level. ARGOS: Argos satellite tags without GPS. ARGOS-GPS: Argos satellite tags with GPS. Iridium: Iridium satellite tags with GPS. DP: days with at least 1 position. ZPI: the interval (in days) with no positions between two consecutive days with at least one position. NPD: number of positions on the same day. NPD_corr_: NPD of ARGOS corrected by duty cycle (see text). ° Only ARGOS satellite tags without duty cycle (i.e., active 24/24 h). * Iridium satellite tags with transmission frequency (IMTI) of max 24 h. NPD > 0: only records with at least 1 position per day. Species: *Cc*, *Caretta caretta*; *Cm*, *Chelonia mydas*; *Lo*, *Lepidochelys olivacea*; *Lk*, *L. kempii*; *Ei*, *Eretmochelys imbricata*. Activity: F, foraging; I, internesting; M, migration. Bayes *p*: Bayesian *p*-value (values near 0 or 1 suggest lack of fit); n/a: not applicable.

	Resp Variable	Categorical Predictors (Reference Level)			Numerical Predictors					
Dataset		Type (Iridium)	Species (*Cc*)	Activity (F)	LAT	MPD	IMTI	Family	N	Bayes *p*
ARGOS °, ARGOS-GPS, Iridium *	DP	uncertain	*Cm+*	I+ M+	+	n/a	n/a	Binomial	13,581	0.40
ARGOS °, ARGOS-GPS, Iridium *	ZPI	ARGOS > ARGOS-GPS	*Lk*−	I−	−	n/a	n/a	Neg Binomial	1094	0.51
ARGOS, ARGOS-GPS, Iridium * (NPD > 0 only)	NPDcorr	uncertain	uncertain	M+	−	n/a	n/a	Neg Binomial	10,147	0.48
Iridium	DP	n/a	*Cm*− *Ei+ Lo+*	M− I−	−	+	uncertain	Binomial	6327	0.62
Iridium	ZPI	n/a	uncertain	I+	uncertain	−	+	Neg Binomial	206	0.56
Iridium (NPD > 0 only)	NPD	n/a	*Cm*−	M− I−	−	+	uncertain	Neg Binomial	5000	0.62

**Table 2:** Number of positions (total *n* = 116,074) obtained during 19,035 monitoring days, by area, species and activity. Species: see Table 1 for abbreviations.

		N Positions (Days)			
Area	Species	Foraging	Internesting	Migration	Total
Caribbean	*Ei*	8436 (761)	80 (13)	816 (115)	9332 (889)
Gulf of Mexico	*Cc*	10,982 (1838)	4890 (166)	1028 (47)	16,900 (2051)
	*Cm*	9227 (1311)	-	-	9227 (1311)
Indian	*Cm*	804 (169)	4307 (223)	231 (40)	5342 (432)
Mediterranean	*Cc*	45,223 (7057)	483 (272)	2547 (302)	48,253 (7631)
NE Pacific	*Lo*	2080 (173)	1839 (255)	-	3919 (428)
NW Atlantic	*Cc*	8762 (3971)	3484 (702)	2745 (862)	14,991 (5535)
	*Cm*	2089 (313)	-	-	2089 (313)
	*Lk*	6021 (445)	-	-	6021 (445)

**Table 3:** Average number of positions per day (NPD) by transmitter type, species and activity, calculated from a total of 116,074 positions obtained during 19,035 monitoring days. Species: see Table 1 for abbreviations.

Type	Species	Activity	Days	Positions (N)	NPD	N Turtles
ARGOS	*Cc*	Foraging	4676	16,705	3.57	10
ARGOS	*Cc*	Internesting	308	1117	3.63	4
ARGOS	*Cc*	Migration	467	1897	4.06	6
ARGOS	*Cm*	Foraging	821	9398	11.45	6
ARGOS	*Lk*	Foraging	200	1214	6.07	2
ARGOS	*Lo*	Foraging	72	84	1.17	1
ARGOS	*Lo*	Internesting	180	310	1.72	3
ARGOS-GPS	*Cc*	Foraging	4668	21,487	4.60	10
ARGOS-GPS	*Cc*	Internesting	325	1335	4.11	4
ARGOS-GPS	*Cc*	Migration	428	1602	3.74	6
ARGOS-GPS	*Cm*	Foraging	563	1729	3.07	5
IRIDIUM-GPS	*Cc*	Foraging	3522	26,775	7.60	13
IRIDIUM-GPS	*Cc*	Internesting	507	6405	12.63	10
IRIDIUM-GPS	*Cc*	Migration	316	2821	8.93	13
IRIDIUM-GPS	*Cm*	Foraging	409	993	2.43	6
IRIDIUM-GPS	*Cm*	Internesting	223	4307	19.31	3
IRIDIUM-GPS	*Cm*	Migration	40	231	5.78	3
IRIDIUM-GPS	*Ei*	Foraging	761	8436	11.09	3
IRIDIUM-GPS	*Ei*	Internesting	13	80	6.15	1
IRIDIUM-GPS	*Ei*	Migration	115	816	7.10	3
IRIDIUM-GPS	*Lk*	Foraging	245	4807	19.62	2
IRIDIUM-GPS	*Lo*	Foraging	101	1996	19.76	2
IRIDIUM-GPS	*Lo*	Internesting	75	1529	20.39	2

## Data Availability

The original contributions presented in this study are included in the article/Supplementary Material. Further inquiries can be directed to the corresponding author.

## Supplementary Materials

The following supporting information can be downloaded at: https://www.mdpi.com/article/10.3390/ani15243605/s1, Table S1: Description of 48 sea turtles tracked by satellite tags of three different types.
