# Supplementary material for: Performance Comparison of Argos and Iridium Tracking Technologies for Sea Turtle Movement Ecology Studies
Source: Animals (Basel). 2025 Dec 15;15(24):3605. doi: 10.3390/ani15243605 (PMC12729580; doi:10.3390/ani15243605)
Supplement: Supplementary file 1 [file animals-15-03605-s001.zip › animals-3990103-supplementary.pdf]

SUPPLEMENTAL MATERIALS

Performance comparison of Argos and Iridium tracking technologies for sea turtle movement ecology studies

Paolo Casale[1*], Christine Figgener[2], Michael Arendt[3], Annette C. Broderick[4], Simona A. Ceriani[5], Yakup Kaska[6], Pamela Plotkin[7], Cheryl L. Sanchez[5], Jeffrey Schwenter[3], Robin Snape[8], Doğan Sözbilen[9], Natalie Wildermann[10], Paolo Luschi[1]

[1]Department of Biology, University of Pisa, Via A. Volta 6, 56126 Pisa, Italy
[2]Namaka Conservation Science, Gandoca, Sixaoala, Talamanca, Limon, Costa Rica
[3]Department of Natural Resources, Marine Resources Division, Charleston, South Carolina, USA
[4]Centre for Ecology and Conservation, University of Exeter, Penryn Campus, Cornwall, UK
[5]Fish and Wildlife Research Institute, Florida Fish and Wildlife Conservation Commission, St. Petersburg FL 33701, USA
[6]Department of Biology, Faculty of Science, University of Pamukkale, Pamukkale, Denizli, Türkiye
[7]Department of Oceanography, Texas A&M University, College Station, Texas, USA
[8]Society for the Protection of Turtles (SPOT), Barbaros sok (23/2), Gönyeli, North Cyprus
[9]Department of Veterinary, Acıpayam Vocational School, Pamukkale University, Denizli, Türkiye
[10]Marine Science Program, Division of Biological and Environmental Science and Engineering, King Abdullah University of Science and Technology, Thuwal, Saudi Arabia.

*Corresponding: paolo.casale@unipi.it

Table S1. Description of 48 sea turtles tracked by satellite tags of three different types, with the number of positions received, the mean number of positions per day (NPD) and main satellite tag settings: duty cycle (DC), maximum number of positions per day (MPD; GPS satellite tags only), and Iridium maximum transmission interval (IMTI). Settings 1 and 2 differentiate periods with different settings within the same turtle track, but are not consistent across turtles. Argos satellite tags: TAM-4310-3 (Telonics, Inc.; turtles 24, 39,42-46), MK10 (Wildlife Computers; turtles 12-15), F6G 276F (Lotek; turtles 26-30), SPLASH (Wildlife Computers; turtles 6-11). Iridium satellite tags were SeaTrkr-4370-4 (Telonics Inc.). §Fastloc®; *FastGPS *Cc: Caretta caretta, Cm: Chelonia mydas; Lk: Lepidochelys kempii, Lo: Lepidochelys olivacea; Ei: Eretmochelys imbricata.* CCL: curved carapace length (cm).

Turtle #	Species	CCL	Stage	Origin	Area	Deployment	Days	Transmitter type	Settings	Activity	N pos	N days	mean NPD	DC	MPD (GPS)	IMTI (Iridium)
1	Cc	74	Adult	Nesting	Turkey (Mediterranean)	13/06/2018	454	IRIDIUM-GPS	1	Foraging	3705	124	29.88	24	48	48
1								IRIDIUM-GPS	2	Foraging	1393	314	4.44	24	8	48
1								IRIDIUM-GPS	1	Migration	278	16	17.38	24	48	48
2	Cc	79	Adult	Nesting	Turkey (Mediterranean)	07/05/2020	395	IRIDIUM-GPS	1	Foraging	351	249	1.41	24	24	24
2								IRIDIUM-GPS	1	Internesting	24	40	0.60	24	24	24
2								IRIDIUM-GPS	1	Migration	123	106	1.16	24	24	24
3	Cc	78	Adult	Nesting	Turkey (Mediterranean)	07/05/2020	472	IRIDIUM-GPS	1	Foraging	3617	406	8.91	24	24	24
3								IRIDIUM-GPS	1	Internesting	54	59	0.92	24	24	24
3								IRIDIUM-GPS	1	Migration	11	7	1.57	24	24	24
4	Cc	85	Adult	Nesting	Turkey (Mediterranean)	08/05/2020	192	IRIDIUM-GPS	1	Foraging	1221	65	18.78	24	24	24
4								IRIDIUM-GPS	1	Internesting	227	113	2.01	24	24	24
4								IRIDIUM-GPS	1	Migration	111	14	7.93	24	24	24
5	Cc	83	Adult	Nesting	Turkey (Mediterranean)	10/05/2020	197	IRIDIUM-GPS	1	Foraging	1401	124	11.30	24	24	24
5								IRIDIUM-GPS	1	Internesting	178	60	2.97	24	24	24
5								IRIDIUM-GPS	1	Migration	137	13	10.54	24	24	24
6	Cc	76 §	Adult	Bycatch	Cyprus (Mediterranean)	02/03/2018	391	ARGOS	1	Foraging	1705	391	4.36	24		
6								ARGOS-GPS§	1	Foraging	2363	389	6.07	24	30	
7	Cc	71 §	Adult	Bycatch	Cyprus (Mediterranean)	13/03/2019	783	ARGOS	1	Foraging	3737	728	5.13	24		
7								ARGOS	1	Migration	745	55	13.55	24		

7								ARGOS-GPS[§]	1	Foraging	5314	725	7.33	24	30
7								ARGOS-GPS[§]	1	Migration	707	55	12.85	24	30
8	Cc	63 §	Juvenile	Bycatch	Cyprus (Mediterranean)	14/04/2019	623	ARGOS	1	Foraging	3361	623	5.39	24	
8								ARGOS-GPS[§]	1	Foraging	5299	622	8.52	24	30
9	Cc	68 §	Juvenile	Bycatch	Cyprus (Mediterranean)	10/05/2019	627	ARGOS	1	Foraging	2792	627	4.45	24	
9								ARGOS-GPS[§]	1	Foraging	2857	625	4.57	24	30
10	Cc	63 §	Juvenile	Bycatch	Cyprus (Mediterranean)	03/02/2018	480	ARGOS	1	Foraging	2014	480	4.20	24	
10								ARGOS-GPS[§]	1	Foraging	3154	481	6.56	24	30
11	Cc	72.1 §§	Adult	Bycatch	Cyprus (Mediterranean)	29/05/2019	61	ARGOS	1	Foraging	398	43	9.26	24	
11								ARGOS	1	Migration	205	18	11.39	24	
11								ARGOS-GPS[§]	1	Foraging	541	41	13.20	24	30
11								ARGOS-GPS[§]	1	Migration	230	18	12.78	24	30
12	Cc	113	Adult	Nesting	Florida (Atlantic)	08/05/2009	733	ARGOS	1	Foraging	310	432	0.72	24	
12								ARGOS	1	Internesting	394	91	4.33	24	
12								ARGOS	1	Migration	228	210	1.09	24	
12								ARGOS-GPS[§]	1	Foraging	463	438	1.06	24	4
12								ARGOS-GPS[§]	1	Internesting	464	104	4.46	24	4
12								ARGOS-GPS[§]	1	Migration	238	189	1.26	24	4
13	Cc	105.1	Adult	Nesting	Florida (Atlantic)	22/05/2009	739	ARGOS	1	Foraging	1382	625	2.21	24	
13								ARGOS	1	Internesting	248	81	3.06	24	
13								ARGOS	1	Migration	48	33	1.45	24	
13								ARGOS-GPS[§]	1	Foraging	885	627	1.41	24	4
13								ARGOS-GPS[§]	1	Internesting	311	81	3.84	24	4
13								ARGOS-GPS[§]	1	Migration	77	29	2.66	24	4
14	Cc	101.3	Adult	Nesting	Florida (Atlantic)	28/05/2009	486	ARGOS	1	Foraging	351	378	0.93	24	
14								ARGOS	1	Internesting	272	81	3.36	24	
14								ARGOS	1	Migration	27	27	1.00	24	
14								ARGOS-GPS[§]	1	Foraging	109	368	0.30	24	4
14								ARGOS-GPS[§]	1	Internesting	362	85	4.26	24	4
14								ARGOS-GPS[§]	1	Migration	6	21	0.29	24	4

15	Cc	110	Adult	Nesting	Florida (Atlantic)	12/05/2009	528	ARGOS	1	Foraging	655	349	1.88	24		
15								ARGOS	1	Internesting	203	55	3.69	24		
15								ARGOS	1	Migration	644	124	5.19	24		
15								ARGOS-GPS§	1	Foraging	502	352	1.43	24	4	
15								ARGOS-GPS§	1	Internesting	198	55	3.60	24	4	
15								ARGOS-GPS§	1	Migration	344	116	2.97	24	4	
16	Cc	85.6	Adult	Nesting	Florida (GoM)	15/07/2020	383	IRIDIUM-GPS	1	Foraging	1399	40	34.98	24	48	48
16								IRIDIUM-GPS	2	Foraging	1454	336	4.33	24	8	48
16								IRIDIUM-GPS	1	Migration	159	7	22.71	24	48	48
17	Cc	83.7	Adult	Nesting	Florida (GoM)	29/05/2021	352	IRIDIUM-GPS	1	Foraging	1548	45	34.40	24	48	48
17								IRIDIUM-GPS	2	Foraging	1839	258	7.13	24	8	48
17								IRIDIUM-GPS	1	Internesting	1481	42	35.26	24	48	48
17								IRIDIUM-GPS	1	Migration	251	7	35.86	24	48	48
18	Cc	97.7	Adult	Nesting	North Carolina (Atlantic)	05/06/2022	77	IRIDIUM-GPS	1	Foraging	138	25	5.52	24	48	48
18								IRIDIUM-GPS	1	Internesting	164	40	4.10	24	48	48
18								IRIDIUM-GPS	1	Migration	31	12	2.58	24	48	48
19	Cc	94	Adult	Nesting	Florida (GoM)	13/05/2021	462	IRIDIUM-GPS	1	Foraging	831	30	27.70	24	48	48
19								IRIDIUM-GPS	2	Foraging	1010	352	2.87	24	8	48
19								IRIDIUM-GPS	1	Internesting	1991	72	27.65	24	48	48
19								IRIDIUM-GPS	1	Migration	106	8	13.25	24	48	48
20	Cc	108	Adult	Nesting	Florida (GoM)	11/07/2021	403	IRIDIUM-GPS	1	Foraging	823	30	27.43	24	48	48
20								IRIDIUM-GPS	2	Foraging	512	352	1.45	24	8	48
20								IRIDIUM-GPS	1	Migration	466	21	22.19	24	48	48
21	Cc	96.7	Adult	Nesting	North Carolina (Atlantic)	04/07/2021	411	IRIDIUM-GPS	1	Foraging	597	25	23.88	24	48	48
21								IRIDIUM-GPS	2	Foraging	1527	277	5.51	24	8	48
21								IRIDIUM-GPS	1	Internesting	449	13	34.54	24	48	48
21								IRIDIUM-GPS	1	Migration	626	22	28.45	24	48	48
21								IRIDIUM-GPS	2	Migration	406	74	5.49	24	8	48
22	Cc	83.4	Adult	Nesting	Florida (GoM)	27/05/2021	451	IRIDIUM-GPS	1	Foraging	1055	40	26.38	24	48	48
22								IRIDIUM-GPS	2	Foraging	511	355	1.44	24	8	48

22								IRIDIUM-GPS	1	Internesting	1418	52	27.27	24	48	48
22								IRIDIUM-GPS	1	Migration	46	4	11.50	24	48	48
23	Cc	105	Adult	Nesting	Florida (Atlantic)	17/05/2022	96	IRIDIUM-GPS	1	Foraging	1843	75	24.57	24	48	48
23								IRIDIUM-GPS	1	Internesting	419	16	26.19	24	48	48
23								IRIDIUM-GPS	1	Migration	70	5	14.00	24	48	48
24	Cm	50.5	Juvenile	Rehab	Florida (Atlantic)	04/11/2020	258	ARGOS	1	Foraging	2066	258	8.01	24		
25	Cm	51.2	Juvenile	Rehab	Florida (Atlantic)	20/07/2021	55	IRIDIUM-GPS	1	Foraging	23	55	0.42	24	24	48
26	Cm	35.7	Juvenile	Capture	Texas (GoM)	03/04/2021	104	ARGOS	1	Foraging	1825	104	17.55	15		
26								ARGOS-GPS*	1	Foraging	391	104	3.76	15	6	
27	Cm	36	Juvenile	Capture	Texas (GoM)	03/04/2021	140	ARGOS	1	Foraging	1555	140	11.11	15		
27								ARGOS-GPS*	1	Foraging	349	140	2.49	15	6	
28	Cm	34.1	Juvenile	Capture	Texas (GoM)	04/10/2020	139	ARGOS	1	Foraging	1316	139	9.47	15		
28								ARGOS-GPS*	1	Foraging	295	139	2.12	15	6	
29	Cm	45.4	Juvenile	Capture	Texas (GoM)	14/07/2021	74	ARGOS	1	Foraging	1043	74	14.09	15		
29								ARGOS-GPS*	1	Foraging	324	74	4.38	15	6	
30	Cm	33.9	Juvenile	Capture	Texas (GoM)	10/07/2021	106	ARGOS	1	Foraging	1593	106	15.03	15		
30								ARGOS-GPS*	1	Foraging	370	106	3.49	15	6	
31	Cm	33.5	Juvenile	Capture	Texas (GoM)	05/04/2021	19	IRIDIUM-GPS	1	Foraging	0	19	-	24	24	96
32	Cm	37.6	Juvenile	Capture	Texas (GoM)	23/11/2019	166	IRIDIUM-GPS	1	Foraging	166	166	1.00	24	24	96
33	Cm	108.9	Adult	Nesting	Aldabra (Indian)	28/01/2022	178	IRIDIUM-GPS	2	Foraging	216	70	3.09	24	24	24
33								IRIDIUM-GPS	1	Internesting	2207	92	23.99	24	48	24
33								IRIDIUM-GPS	1	Migration	65	15	4.33	24	48	24
33								IRIDIUM-GPS	2	Migration	5	1	5.00	24	24	24
34	Cm	108.4	Adult	Nesting	Aldabra (Indian)	29/01/2022	81	IRIDIUM-GPS	1	Foraging	10	2	5.00	24	48	24
34								IRIDIUM-GPS	2	Foraging	23	4	5.75	24	24	24
34								IRIDIUM-GPS	1	Internesting	435	65	6.69	24	48	24
34								IRIDIUM-GPS	1	Migration	38	10	3.80	24	48	24
35	Cm	107	Adult	Nesting	Aldabra (Indian)	28/01/2022	173	IRIDIUM-GPS	2	Foraging	555	93	5.97	24	24	24
35								IRIDIUM-GPS	1	Internesting	1665	66	25.23	24	48	24
35								IRIDIUM-GPS	1	Migration	75	8	9.38	24	48	24

35								IRIDIUM-GPS	2	Migration	48	6	8.00	24	24	24
36	Ei	85.6	Adult	Nesting	Costa Rica (Carib)	22/09/2020	168	IRIDIUM-GPS	1	Foraging	1181	112	10.54	24	12	48
36								IRIDIUM-GPS	1	Internesting	80	13	6.15	24	12	48
36								IRIDIUM-GPS	1	Migration	242	43	5.63	24	12	48
37	Ei	89.9	Adult	Nesting	Costa Rica (Carib)	06/09/2021	372	IRIDIUM-GPS	1	Foraging	3722	336	11.08	24	12	48
37								IRIDIUM-GPS	1	Migration	270	36	7.50	24	12	48
38	Ei	86.7	Adult	Nesting	Costa Rica (Carib)	16/10/2021	349	IRIDIUM-GPS	1	Foraging	3533	313	11.29	24	12	48
38								IRIDIUM-GPS	1	Migration	304	36	8.44	24	12	48
39	Lk	62	Adult	Capture	South Carolina (Atlantic)	09/10/2017	122	ARGOS	1	Foraging	710	122	5.82	24		
40	Lk	64	Adult	Capture	South Carolina (Atlantic)	18/07/2018	71	IRIDIUM-GPS	1	Foraging	1288	71	18.14	24	24	48
41	Lk	64.2	Adult	Capture	Georgia (Atlantic)	13/07/2017	174	IRIDIUM-GPS	1	Foraging	3519	174	20.22	24	24	48
42	Lk	>57.4	Adult	Capture	Florida (Atlantic)	18/07/2018	78	ARGOS	1	Foraging	504	78	6.46	24		
43	Lo	64.7	Adult	Nesting	Costa Rica (Pacific)	27/08/2017	55	ARGOS	1	Internesting	87	55	1.58	2.4		
44	Lo	62.1	Adult	Nesting	Costa Rica (Pacific)	24/08/2017	72	ARGOS	1	Foraging	84	72	1.17	2.4		
45	Lo	68.7	Adult	Nesting	Costa Rica (Pacific)	13/12/2017	45	ARGOS	1	Internesting	77	45	1.71	2.4		
46	Lo	67.8	Adult	Nesting	Costa Rica (Pacific)	21/12/2017	80	ARGOS	1	Internesting	146	80	1.83	2.4		
47	Lo	65.5	Adult	Nesting	Costa Rica (Pacific)	06/08/2017	119	IRIDIUM-GPS	1	Foraging	1550	80	19.38	24	24	48
47								IRIDIUM-GPS	1	Internesting	770	39	19.74	24	24	48
48	Lo	63.3	Adult	Nesting	Costa Rica (Pacific)	17/08/2017	57	IRIDIUM-GPS	1	Foraging	446	21	21.24	24	24	48
48								IRIDIUM-GPS	1	Internesting	759	36	21.08	24	24	48
